# The effects of dark chocolate on cognitive performance during cognitively demanding tasks: A randomized, single-blinded, crossover, dose-comparison study

**DOI:** 10.1016/j.heliyon.2024.e24430

**Published:** 2024-01-11

**Authors:** Akihiro Sasaki, Kei Mizuno, Yusuke Morito, Chisato Oba, Kentaro Nakamura, Midori Natsume, Kyosuke Watanabe, Emi Yamano, Yasuyoshi Watanabe

**Affiliations:** a Laboratory for Pathophysiological and Health Science, RIKEN Center for Biosystems Dynamics Research, Kobe, Japan; b RIKEN Compass to Healthy Life Research Complex Program, Kobe, Japan; c Center for Health Science Innovation, Osaka Metropolitan University, Osaka, Japan; d Food Microbiology Research Laboratories, R&D Division, Meiji Co., Ltd., Hachioji, Tokyo, Japan

**Keywords:** Cacao polyphenols, Chocolate, Cognitive function, Concentration, Fatigue

## Abstract

Dark chocolate, rich in polyphenols, increases cerebral blood flow and improves cognitive function. This study aimed to determine whether the consumption of chocolate with a high concentration of polyphenols helps to maintain cognitive performance during cognitively demanding tasks. In this randomized, single-blinded, crossover, dose-comparison study, 18 middle-aged adults consumed two types of chocolate (25 g each), one with a high concentration (635.0 mg) and the other with a low concentration (211.7 mg) of cacao polyphenols, and performed a cognitive task requiring response inhibition and selective attention over two time periods (15–30 min and 40–55 min after consumption, respectively). Autonomic nerve function and subjective feelings, such as fatigue and concentration, were measured before food intake and after the second task to assess the participant's state. The results showed that the average reaction time between the first and second sessions was not significantly different for either high- or low-concentration chocolate consumption. However, the percentage of correct responses was similar in the first (96.7 %) and second (96.8 %) sessions for high-concentration chocolate consumption and significantly lower for low-concentration chocolate consumption in the second (96.4 %) session than in the first session (97.3 %). Autonomic nerve function showed a significant increase in sympathetic nerve activity after the second task with high-concentration chocolate consumption, while subjective feelings showed an increase in mental fatigue for both chocolate types but a significant decrease in concentration only after the second task with low-concentration chocolate consumption. These findings suggest that dark chocolate consumption contributes to the maintenance of performance and concentration in continuous and demanding cognitive tasks.

## Highlights

• Dark chocolate, rich in polyphenols, is known to increase cerebral blood flow and has been suggested to improve cognitive function.
• In this study, 18 middle-aged adults consumed two types of chocolate, one with high (635.0 mg) and the other with low (211.7 mg) cacao polyphenols (HC and LC chocolate, respectively), and performed a cognitive task.
• In two consecutive sessions, the percentage of correct responses significantly decreased in the second session compared to the first for LC chocolate consumption, whereas it was maintained for HC chocolate consumption.
• Our findings suggest that consumption of dark chocolate contributes to the maintenance of performance and concentration in a continuously and effortfully cognitive task.

## 1 Introduction

Chocolate is one of the most widely consumed confectioneries worldwide. Particularly, dark chocolate has a higher polyphenol content than foods such as red wine, coffee, and apples [1]. Cacao liquor, the main ingredient in chocolate, contains abundant methylxanthines and polyphenols. Methylxanthines, including theobromine and caffeine, stimulate the central nervous system [2,3]. Cacao polyphenols, including epicatechin, catechin, and procyanidins [4], have antioxidant properties [5] and improve endothelial vascular function [[6], [7], [8]].

Recent studies have shown that cacao polyphenols increase cerebral blood flow, which is associated with improved cognitive function. For example, continuous cacao polyphenol consumption for 3 months increased cerebral blood volume in the dentate gyrus, and this increase was positively correlated with improved memory performance [9]. An increase in cerebral blood flow in the anterior cingulate gyrus and central opercular cortex of the left parietal lobe has also been observed even after acute ingestion of cacao polyphenols [10]. The increase in cerebral blood flow with acute consumption is transient, as indicated by a preliminary study of a small number of participants in their 30s, where a single 900-mg dose of cacao polyphenols reached peak cerebral blood flow approximately 2 h after ingestion and returned to baseline approximately 6 h after ingestion [11].

There is a known association between habitual chocolate consumption and cognitive function [12]. The effect of continuous cacao polyphenol intake on cognitive performance has also been reported [13]. However, the effects of single consumption of cacao polyphenol drink and cacao polyphenol-enriched chocolate on cognitive function have not been fully established. Some studies have shown that a single intake of cacao polyphenol-fortified chocolate improved cognitive performance [14,15], while another study has found no improvement [16,17]. Alongside the effects of sleep deprivation on cognitive decline [18], the positive effects of cacao consumption on performance and fatigue in continuous subtraction tasks [19] suggest that these effects may occur under physically or mentally demanding conditions.

This study aimed to determine whether the consumption of dark chocolate with high concentrations of cacao polyphenols helps maintain cognitive performance during cognitively demanding tasks. To accomplish this, this study used a “traffic light test,” which requires response inhibition to a Stroop stimulus [20] and selective attention to a target stimulus while ignoring a disturbing stimulus. Participants consumed two types of chocolate, one with a high cacao concentration and the other with a low cacao concentration, and performed cognitive tasks requiring response inhibition and selective attention over two time periods. Subsequently, the changes in cognitive performance during the two time periods were examined.

## 2 Materials and methods

### 2.1 Participants

**Fig. 1:**
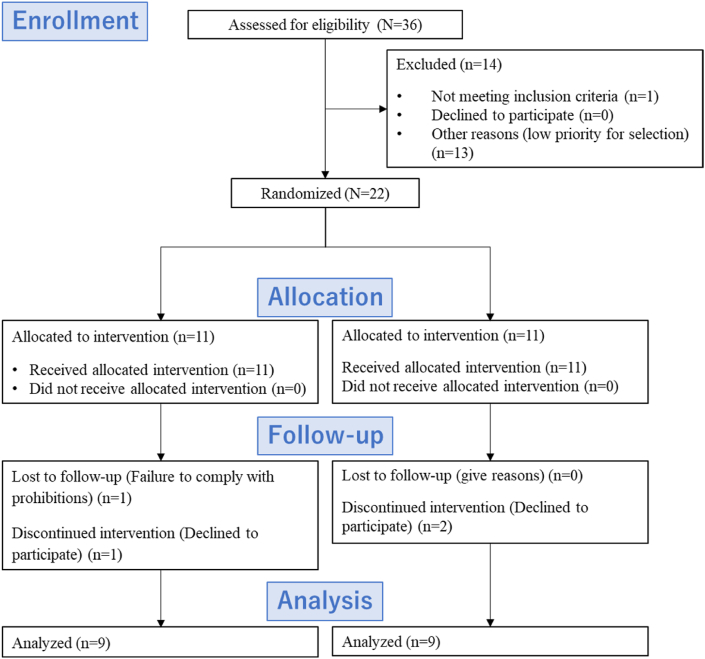
Participant flowchart.

Overall, 36 adult healthy volunteers (17 males and 19 females, aged 30–49 years) participated in this study. For the screening test, we initially selected 35 of the 36 participants who met the inclusion and exclusion criteria (Table 1). Of these 35 participants, 22 were selected in the order of earliest entry into the study, allowing for an equal number of males and females. Finally, 22 participants (11 males and 11 females) participated in the main experiment (Fig. 1). The mean age was 35.4 ± 8.2 years; the mean body mass index (BMI) was 20.8 ± 1.9 kg/m^2^. The study was conducted in compliance with national legislation and the Code of Ethical Principles for Medical Research Involving Human Participants of the World Medical Association (Declaration of Helsinki) and was registered in the University Hospital Medical Information Network (UMIN) Clinical Trials Registry (No. UMIN000036746). The protocol was approved by the Ethics Committees of the RIKEN Center for Biosystems Dynamics Research (RIKEN-K2-2018-09) and Meiji Co., Ltd. (No. 165). All participants were informed about the study and provided written informed consent before screening.

**Table 1:** List of the inclusion and exclusion criteria.

Inclusion criteria	
1	Individuals who have had the purpose and content of this study fully explained, understood them, and given their informed consent in writing of their free will.
2	Healthy men and women between the ages of 30 and 49 at the time consent is obtained.
Exclusion criteria	
1	Persons who have consumed pharmaceuticals, quasi-drugs, or dietary supplements at least 5 days per week during the month before the screening test (SCR) or plan to do consume them during the study period
2	Persons who have or have had serious diseases, such as cranial nerve disease, liver disease, kidney disease, cardiac disease, circulatory disease, or malignant tumors
3	Persons who have or have had seizures (loss of consciousness, coma, convulsions due to neurological diseases)
4	Persons who have a problem with the measurement of autonomic nerve function (LF/HF ≥ 10)
5	Persons with food allergies
6	Persons who cannot consume bitter chocolate (dark chocolate)^a^
7	Persons who consume excessive alcohol (average of 60 g/day or more)
8	Habitual smokers
9	Persons who work day/night shifts and those who work night shifts during the study period
10	Persons who have extremely poor performance on the cognitive function test during the SCR
11	Persons who have participated in another clinical research study within 1 month before providing consent to participate in this study or plan to participate in another clinical research study during the study period after providing consent to participate in this study
12	Persons who are pregnant or possibly pregnant, lactating, or planning or hoping to become pregnant during the study period
13	Persons who are judged to be inappropriate as a participant by the Principal Investigator or a Research Assigning Physician

SCR, screening test.

a Bitter (dark) chocolate is defined as chocolate with 40–60 % cocoa liquor without milk (dairy), chocolate with low sugar and dairy content, high bitterness, or low sugar chocolate with 70–90 % cocoa content.

### 2.2 Test foods

Two types of chocolate containing different cacao polyphenols were used in this study (Table 2). Both test foods were manufactured by the R&D division of Meiji Co., Ltd. (Hachioji, Japan). The contents of each chocolate were known only to the manufacturers; therefore, the allocation was concealed from the investigators. Test food 1 contained chocolate with a high concentration of polyphenols (HC) (635.0 mg of cacao polyphenol). Test food 2 contained chocolate with a low concentration of polyphenols (LC) (211.7 mg of cacao polyphenols). The HC chocolate had the same composition as that used in a previous study, which showed that 25 g of chocolate per day for 4 weeks increased brain-derived neurotrophic factor, high-density lipoprotein cholesterol, and quality of life scores and decreased blood pressure [21]. However, the LC chocolate was prepared by replacing cacao liquor with dextrin and cellulose to minimize the amount of cacao liquor and preserve the properties of the chocolate. The polyphenol content of the test foods was analyzed using Folin–Ciocalteu methods [22] with (−)-epicatechin (SIGMA Chemical CO, St. Louis, MO, USA) as the standard. The procyanidin, caffeine, and theobromine contents were quantified in a high-performance liquid chromatography analysis [4]. It contained a lower effective amount of cacao polyphenols than that reported in previous studies. White chocolate was not used as a control food to avoid blinding due to its appearance and the side effects of milk protein. This dose-contrast design for cacao polyphenols was used to control energy intake and to make participants believe that they were consuming dark chocolate when they consumed either test food based on appearance and taste.

**Table 2:** Ingredients in high polyphenol concentration chocolate and low polyphenol concentration chocolate.

	High concentration cacao chocolate	Low concentration cacao chocolate
Color/Shape	Brown/Plate	
Amount (per test) (g)	25.0 ± 1.0	
Energy (kcal)	131.7	138.5
Fat-free cacao content (g)	8.7	2.9
Total polyphenol (mg)	635.0	211.7
Procyanidins^a^	67.5	22.2
Epicatechin (mg)	30.4	10.5
catechin (mg)	8.5	3.3
procyanidin B2 (mg)	12.3	4.3
procyanidin B5 (mg)	2.9	0.5
procyanidin C1 (mg)	8.3	3.2
cinnamtannin A2 (mg)	5.1	0.5
Theobromine (mg)	200.0	66.7
Caffeine (mg)	20.0	6.7

a Mainly components of cacao polyphenols (show as total amount of catechin, epicatechin, procyanidin B2, procyanidin B5, procyanidin C1, and cinnamtannin A2).

### 2.3 Study design

**Fig. 2:**
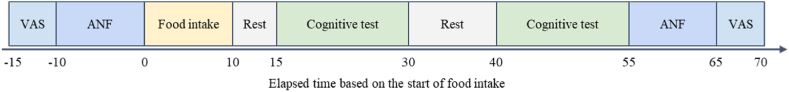
Gantt timeline showing the daily study schedule ANF, measurement of autonomic nerve function; VAS, visual analog scale.

This single-blind (investigator- and assessor-blinded) randomized dose-comparison study with a two-phase crossover design was conducted at RIKEN BDR between April 2019 and June 2019. Twenty-two participants enrolled in the main experiment were randomly allocated to one of the two groups (Fig. 1). Group A consumed HC and LC chocolates in Trials 1 and 2, respectively, whereas Group B consumed LC and HC chocolates in Trials 1 and 2, respectively. An allocation manager randomly allocated the participants to either Group A or Group B, using stratified randomization to match the age and sex ratios of the two groups (Fig. 1). The allocation table with each participant's ID number and test food code was prepared by the allocation manager and subsequently sealed until the test food representative unblinded the codes after the data were finalized at the case conference. Thus, both the investigators and assessor remained blinded throughout the study period, ensuring single-blindness. This study followed the CONSORT guidelines for reporting randomized crossover trials [23]. Trials 1 and 2 were conducted 1 week apart as a washout period. Therefore, the participants consumed the test food on 2 separate days, with a 1-week washout period. The 1-day study schedule was as follows (Fig. 2): First, after the participant's physical condition was examined and the schedule was explained, they completed a subjective feeling questionnaire, and their autonomic nerve function was measured. Subsequently, participants ate the full amount (25 g) of the test food during a 10-min test food intake period; after a 5-min rest period, the first cognitive function test was performed for 15 min. After a 10-min rest period, a second cognitive function test was performed for 15 min. Finally, the participants completed the subjective mood questionnaire again, and their autonomic nerve function was measured.

### 2.4 Data acquisition

#### 2.4.1 Cognitive task

We used a modified version of the Stroop task, which comprised a cognitive task presentation involving traffic lights (placed on a letter corresponding to blue or red in Japanese), traffic signs of walkers (right or left), and turns (right or left) displayed on the screen of a laptop computer (Dynabook Satellite B35, Toshiba Co., Ltd., Tokyo) [[24], [25], [26]]. Participants were required to press the left button with their left index finger as soon as the letter meaning red appeared and to press the right button with their right index finger as soon as the letter meaning blue appeared, regardless of whether the light was red or blue, a traffic sign of walkers, or turns. Each trial appeared 100 ms after either button was pressed and was repeated until 15 min had elapsed. In each trial, letters, traffic lights with letters presented, and pedestrian and turn signal traffic signs were presented pseudo-randomly, with the same frequency as Stroop trials where the meaning of the presented letter and the color of the traffic light were mismatched, and non-Stroop trials where the meaning of the presented letter and the color of the traffic light were matched. The participants performed two 15-min sessions (first and second) with 10 min of rest between sessions (Fig. 2).

#### 2.4.2 Autonomic nerve function

To assess autonomic nerve function, the Vital Monitor 302 system (VM302, Fatigue Science Institute, Osaka, Japan) was used to simultaneously measure the electrocardiogram (ECG) and photoplethysmogram (PPG) for 3 min while the participants sat quietly with their eyes closed. The recorded data were analyzed using MemCalc software (GMS, Tokyo, Japan). Frequency analysis for *R*–R interval variation from the ECG and a-a interval variation as the second derivative from the PPG (accelerated plethysmography) were performed using the maximum entropy method, which can estimate the power spectral density from short time series data and is suitable for studying changes in heart rate variability under different conditions for brief periods [27,28]. The power spectrum resolution was 600 Hz. In the frequency analysis, power within the frequency range of 0.04–0.15 Hz was calculated as low frequency (LF), and power within the frequency range of 0.15–0.4 Hz was calculated as high frequency (HF). HF is mediated by the vagus nerve [[29], [30], [31]], whereas LF is derived from various sympathetic and vagal mechanisms [28,32]. Some review articles [24,33,34] have reported that LF reflects sympathetic nerve activity; therefore, it has been used as a marker. To stabilize the measurement, a 1-min pretest was performed before the main 3-min test. The main test was administered twice as follows: before food intake and after the cognitive tasks (Fig. 2).

#### 2.4.3 Subjective ratings

Subjective feelings were measured using a visual analog scale (VAS) for overall fatigue, mental fatigue, physical fatigue, stress, boredom, sleepiness, motivation, relaxation, enjoyment, healing, concentration, and willingness before food intake and after the cognitive tasks (Fig. 2). Participants were instructed to draw a line intersecting the 100-mm horizontal line at the location representing each of the feelings; the left and right ends of the VAS represented the lowest and highest values of each feeling, respectively.

### 2.5 Statistical analysis

#### 2.5.1 Analysis group

The sample size was based on previous behavioral studies with similar designs [25,35], as the effect size was not known in advance upon study initiation. The intention-to-treat (ITT) analysis set was defined as the population of all assigned participants. The per-protocol analysis set (PPS) was defined as participants who completed the full schedule of the study and did not meet any of the following analytic exclusion criteria: intake of the test food less than 90 %, behavior that undermined the reliability of test results (e.g., sleeping during the test), or found after consent to meet the exclusion criteria or not to meet the selection criteria.

#### 2.5.2 Analytic method

Cognitive function was analyzed for the number of correct responses, number of incorrect responses, number of no responses, percentage of correct responses, and average reaction time in all trials and based on trial type. Autonomic nerve function was analyzed using the LF, HF, and LF/HF ratios. Each subjective feeling was quantified by measuring the distance from the left end of the VAS to the position of the line drawn by the participant with an accuracy of 1 mm. The VAS scores ranged from 0 to 100. In this study, we focused on the contrast between rounds 1 and 2 for cognitive function and before and after chocolate consumption for autonomic nerve function and subjective feelings. For statistical testing, we performed multiple comparison tests using Bonferroni correction for each chocolate condition. Paired t-tests were used for cognitive performance and autonomic nerve function, and Wilcoxon signed-rank tests were used for subjective ratings.

### 2.6 Outcome measures

The primary outcomes were cognitive task performances during the period from 5 to 15 min after test food ingestion and from 30 to 45 min after test food ingestion.

The secondary outcomes included subjective feelings and autonomic nervous function before and after test food intake and the number of side effects within 24 h after test food intake.

## 3 Results

### 3.1 Participant information

The analysis included 22 ITT and 18 PP S participants; two of the 22 ITT participants dropped out before the efficacy evaluation. Therefore, statistical analysis for efficacy evaluation was performed on 20 and 18 participants treated with ITT and PPS, respectively. For within-group comparisons under the HC intake condition, the ITT and PPS analysis populations were identical. The mean age, height, weight, and BMI were comparable between Groups A and B (age: t (20) = 0.30, p = 0.765; BMI: t (20) = 0.55, p = 0.590). The sex ratios of participants were also comparable between Groups A and B (χ^2^ = 0.182, p = 0.670). Thus, no bias was found in the participant characteristics (age, BMI, or sex) among the groups with different orders of test food intake. The demographic data and statistical results are summarized in Table 3. The chocolate intake rate for each participant was 100 % on all test days. The number of adverse events and side effects that occurred was 0 during the study period and 24 h after consumption of test food.

**Table 3:** Participant information.

	Group A	Group B	*P*-value
**Intention-to-treat**			
Number of participants (female/male)	11 (5/6)	11 (6/5)	0.670^a^
Mean age ± SD (years)	35.9 ± 9.6	34.8 ± 7.1	0.765^b^
Body mass index ± SD (kg/m^2^)	21.0 ± 1.9	20.6 ± 2.0	0.590^b^
**Per-protocol set**			
Number of participants (female/male)	9 (5/4)	9 (4/5)	0.673^a^
Mean age ± SD (years)	34.4 ± 9.7	35.3 ± 7.3	0.829^b^
Body mass index ± SD (kg/m^2^)	20.9 ± 2.1	20.9 ± 2.0	0.991^b^

a p-values calculated by χ^2^ test.

b p-values calculated by two-sample *t*-test.

### 3.2 Crossover design adequacy

To confirm the adequacy of the crossover design, we compared the mean reaction time and accuracy rate of the PPS group. No significant difference was found in the mean reaction time (t (15) = −1.22, p = 0.24) and accuracy rate (t (15) = −2.07, p = 0.06), indicating that no biases existed between the groups.

### 3.3 Cognitive function

**Fig. 3:**
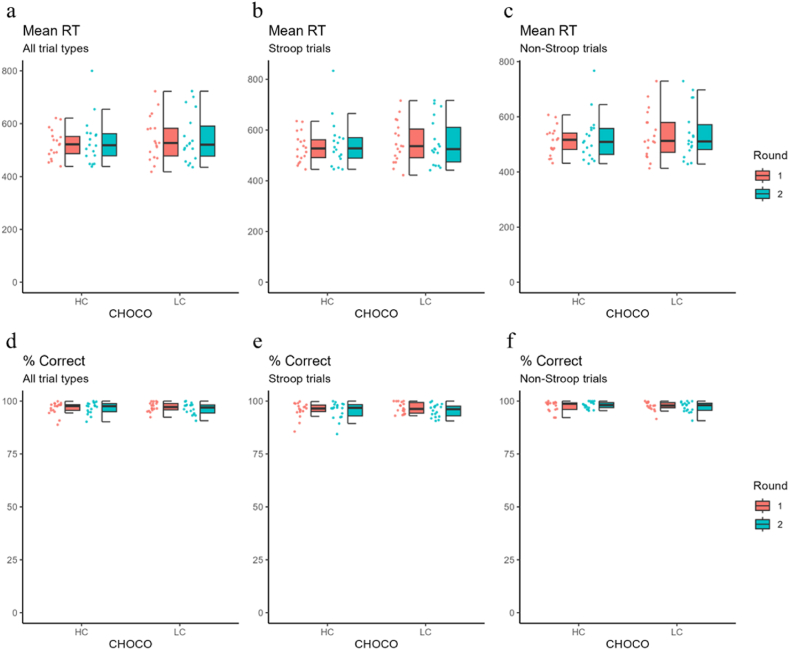
Performance of cognitive function test The top three panels show the average reaction time (RT) on the “traffic light test” for all trials (a), Stroop trials (b), and non-Stroop trials (c), respectively, in dot plots and box charts. The bottom three panels show the percentage of correct responses (%) on the “traffic light test” for all trials (d), Stroop trials (e), and non-Stroop trials (f), respectively, in dot plots and box charts. HC, high concentration cacao chocolate; LC, low concentration cacao chocolate.

Cognitive ability was assessed based on the mean reaction time and percentage of correct responses for all trials, the Stroop trials, and the non-Stroop trials. As shown in Fig. 3 and Table 4, for all trial types, the mean reaction time was slightly longer in round 1 than in round 2 for both HC and LC chocolates; however, these differences were not statistically significant (Ps > 0.4, Fig. 3a–c and Table 4). For all trials, the percentage of correct responses under HC chocolate consumption was comparable for rounds 1 (96.7 ± 2.9 %) and 2 (96.8 ± 2.7 %) (t (17) = 0.34, p = 0.74, Fig. 3d). Conversely, the percentage of correct responses for LC chocolate consumption was significantly lower in round 2 (96.4 ± 2.6 %) than in round 1 (97.3 ± 2.2 %) (t (17) = −2.75, p = 0.014, Fig. 3d). Similarly, for the Stroop trials, the percentage of correct responses under HC chocolate consumption was comparable between rounds 1 (95.8 ± 3.6 %) and 2 (95.4 ± 4.0 %) (t (17) = −0.54, p = 0.597, Fig. 3e), while that under LC chocolate consumption was statistically significantly lower in round 2 (96.7 ± 2.6 %) than in round 1 (95.5 ± 3.0 %) (t (17) = −3.12, p = 0.006, Fig. 3e). For non-Stroop trials, no statistically significant difference was found in the percentage of correct responses between rounds 1 and 2 under either HC (97.5 ± 2.4 % for round 1 and 98.0 ± 1.6 % for round 2; t (17) = 1.17, p = 0.26, Fig. 3f) or LC chocolate consumption (97.8 ± 2.1 % for round 1 and 97.2 ± 2.5 % for round 2; t (17) = −1.81, p = 0.088, Fig. 3f).

**Table 4:** Cognitive function test.

	High polyphenol concentration chocolate: Round 2 - Round 1					Low polyphenol concentration chocolate: Round 2 - Round 1				
	t	df	p	CI (low)	CI (high)	t	df	p	CI (low)	CI (high)
***All trial types (Stroop and non-Stroop trials)***										
Mean RT	0.72	17	0.482	−21.59	43.94	0.12	17	0.904	−19.78	22.22
% correct	0.34	17	0.735	−0.71	0.99	−2.75	17	**0.014**	−1.55	−0.21
***Stroop trials***										
Mean RT	0.70	17	0.495	−24.39	48.51	0.04	17	0.965	−20.13	21.00
% correct	−0.54	17	0.597	−1.28	0.76	−3.12	17	**0.006**	−2.00	−0.39
***Non-Stroop trials***										
Mean RT	0.77	17	0.451	−18.63	40.14	0.22	17	0.826	−19.71	24.38
% correct	1.17	17	0.260	−0.40	1.39	−1.81	17	0.088	−1.31	0.10

Results of paired *t*-test between Round 2 and Round 1. The statistical threshold is p = 0.025, corresponding to p = 0.05 with Bonferroni correction. Bold type indicates statistical significance. CI, confidence interval of 95 %; df, degrees of freedom; RT, reaction time.

### 3.4 Autonomic nerve function

**Fig. 4:**
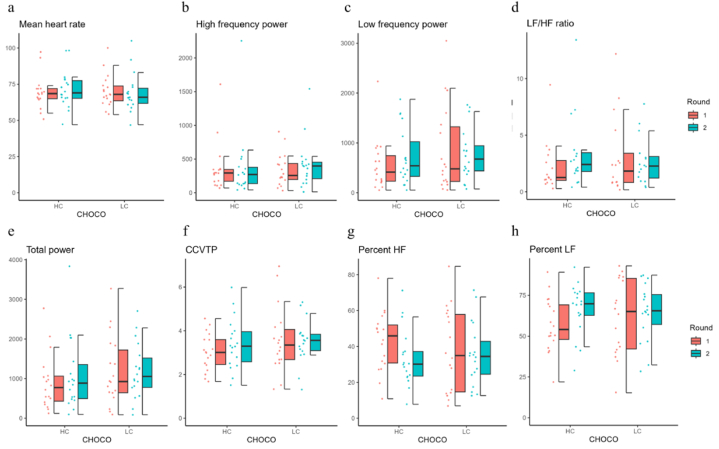
Autonomic nerve function Each panel shows the mean heart rate (a), low-frequency power (LF) (b), high-frequency power (HF) (c), total power (d), LF/HF ratio (e), coefficient of component variance total power (CCVTP) (f), percentage of LF (g), and percentage of HF (h) in dot plots and box charts, respectively. HC, high concentration cacao chocolate; LC, low concentration cacao chocolate.

Autonomic nerve function was assessed based on mean heart rate, LF power, HF power, their ratio (LF/HF), total power (TP), coefficient of component variance total power (CCVTP), and the proportions of LF and HF (percentage of LF and HF, respectively) (Fig. 4 and Table 5). Mean heart rate, LF, HF, TP and LF/HF did not differ significantly between rounds 1 and 2 when either the HC or LC cacao chocolate was consumed (p > 0.07, Fig. 4a–e). The CCVTP and percentage of LF and HF significantly changed with HC cacao chocolate consumption (t (15) = 2.81, p = 0.12, Fig. 4f–h). The percentage of LF was higher in round 2 than in round 1, and that of HF was lower in round 2 than in round 1. This means that LF dominated the balance between LF and HF power in round 2. However, there was no statistically significant change in the proportion of LF and HF in LC chocolate consumption.

**Table 5:** Autonomic nerve function.

	High polyphenol concentration chocolate: Round 2 – Round 1					Low polyphenol concentration chocolate: Round 2 – Round 1				
	t	df	p	CI (low)	CI (high)	t	df	p	CI (low)	CI (high)
Heart rate	2.3	17	0.034	0.17	4.05	−0.95	17	0.356	−3.76	1.43
LF	1.63	17	0.122	−51.09	396.50	−0.49	17	0.631	−389.48	242.93
HF	0.13	17	0.894	−88.32	100.36	1.12	17	0.277	−68.54	224.72
TP	1.33	17	0.202	−105.25	462.67	0.03	17	0.979	−375.12	384.73
LF/HF	1.92	15	0.074	−0.14	2.63	−0.63	15	0.536	−1.59	0.86
ccvTP	2.48	17	**0.024**	0.06	0.72	−0.22	17	0.830	−0.61	0.50
%LF	2.81	17	**0.012**	2.34	16.45	0.96	17	0.348	−3.40	9.14
%HF	−2.81	17	**0.012**	−16.45	−2.34	−0.96	17	0.348	−9.14	3.40

Results of paired *t*-test between Round 2 and Round 1. The statistical threshold is p = 0.025, corresponding to p = 0.05 with Bonferroni correction. Bold type indicates statistical significance. CI, confidence interval of 95 %; df, degrees of freedom. LF, low frequency; HF, high frequency; ccVTP, coefficient of component variance total power.

### 3.5 Subjective ratings

**Fig. 5:**
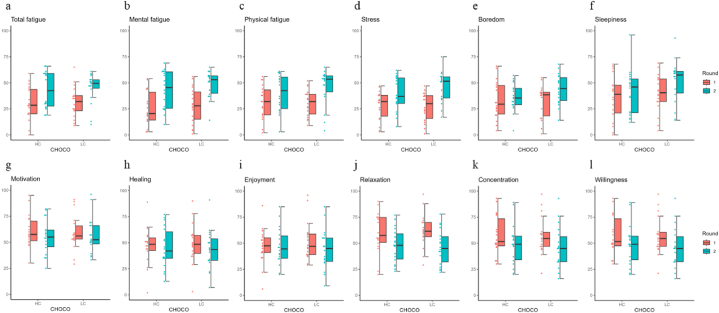
Subjective feelings Each panel shows the value of subjective feelings measured using a visual analog scale. Each panel shows the value of total fatigue (a), mental fatigue (b), physical fatigue (c), stress (d), boredom (e), sleepiness (f), motivation (g), healing (h), enjoyment (i), relaxation (j), concentration (k), and willingness (l), respectively, in dot plots and box charts. HC, high concentration cacao chocolate; LC, low concentration cacao chocolate.

The subjective feelings scored using the VAS are shown in Fig. 5 and Table 6. Total fatigue, mental fatigue, and stress statistically significantly increased in round 2 compared with round 1 under both HC (V = 139, p = 0.020 for total fatigue; V = 150.5, p = 0.005 for mental fatigue; V = 128, p = 0.015 for stress) and LC (V = 150, p = 0.005 for total fatigue; V = 164.5, p = 0.001 for mental fatigue; V = 164, p = 0.001 for stress) chocolate consumption (Fig. 5a,b, and d). Physical fatigue and boredom also increased in round 2 under both HC and LC chocolate consumption; however, statistical significance was found only for LC chocolate consumption (V = 126, p = 0.078 for physical fatigue under HC chocolate; V = 96, p = 0.647 for boredom under HC chocolate; V = 156.5, p = 0.002 for physical fatigue under LC chocolate; and V = 137.5, p = 0.023 for boredom under LC chocolate Fig. 5c and e). Consistent with these results, we found that in round 2, the HC and LC chocolate groups were significantly less relaxed than they were in round 1 (V = 25, p = 0.015 for HC chocolate; V = 12, p = 0.001 for LC chocolate, Fig. 5j). The concentration level was also decreased in round 2 compared with round 1 for both HC and LC chocolate; however, this change was only statistically significant for LC chocolate consumption (V = 44, p = 0.124 for HC chocolate; V = 13, p = 0.003 for LC chocolate, Fig. 5k). Sleepiness, motivation, healing, enjoyment, and willingness did not change significantly for either HC or LC chocolate consumption (Fig. 5f–i, and l).

**Table 6:** Subjective feelings.

	High-concentration of polyphenols (HC) chocolate: Round 2 － Round 1				Low-concentration of polyphenols (LC) chocolate: Round 2 － Round 1			
	t	p	CI (low)	CI (high)	t	p	CI (low)	CI (high)
Total fatigue	139	**0.020**	2.5	24	150	**0.005**	5.5	22
Mental fatigue	150.5	**0.005**	8	27.5	164.5	**0.001**	11.5	28.5
Physical fatigue	126	0.078	−2	20	156.5	**0.002**	9.5	23
Stress	128	**0.015**	4	22	164	**0.001**	12.5	28
Boredom	96	0.647	−9	15	137.5	**0.023**	1.5	21
Sleepiness	106	0.372	−6	16	125.5	0.081	−1.5	23.5
Motivation	41	0.162	−14.5	2	65	0.371	−9	4
Healing	61	0.286	−9	4.5	44.5	0.13	−14	1.5
Enjoyment	81	0.844	−6	6	36	0.098	−17	1
Relaxation	25	**0.015**	−22.5	−3.5	12	**0.001**	−23	−7
Concentration	44	0.124	−20.5	2	13	**0.003**	−18	−4.5
Willingness	36.5	0.103	−16	4	37.5	0.036	−15	−0.5

Results of Wilcoxon signed rank test between Round 2 and Round 1. The statistical threshold is p = 0.025, corresponding to p = 0.05 with Bonferroni correction. Bold type indicates statistical significance. CI, confidence interval of 95 %.

## 4 Discussion

This study examined the effects of consuming HC chocolate on cognitive exertion regarding cognitive performance, autonomic nerve function, and subjective feelings compared with consuming LC chocolate. We found that when LC chocolate was consumed, there was a lower percentage of correct responses to the second half (round 2) of the trials overall and of the Stroop trials than the first half (round 1), though this was not observed in the performance of the non-Stroop trials. No significant decrease was observed when HC chocolate was consumed. Regarding reaction time, we found no significant differences between the first and second halves for any trial type when either the LC or HC chocolate was consumed.

### 4.1 Maintained cognitive function for demanding tasks

Notably, the percentage of correct responses in the cognitive task was maintained with HC chocolate consumption and decreased with LC chocolate consumption. The “traffic light test” used in this study is a task that utilizes cognitive conflict due to mismatches between color name words and background colors (the Stroop effect) and suppression of attention to interfering stimuli, requiring response inhibition and selective attention, respectively. Thus, this task required significant cognitive effort from participants. Poor performance in cognitively demanding continuous tasks is frequently associated with fatigue or fatigue due to sleep loss, as previous studies have shown that acute mental fatigue increases error rates in traffic light tests [24,35] and Stroop tasks [36,37]. Particularly, fatigue due to a prolonged cognitive load has been demonstrated to impair attention control [38] and inhibit attention to irrelevant information [39], suggesting that fatigue can affect attentional performance. It is also noteworthy that reaction times to cognitive tasks did not differ significantly between the HC and LC chocolate consumption groups. A previous study investigated the effects of mental fatigue on the duration of actual goal-directed and imaginative arm movements with a speed-accuracy trade-off, suggesting that in the presence of mental fatigue, movements are slower because of proactive changes that occur in the readiness state of the movement to maintain task success [40]. Overall, it is possible that reaction time was prioritized over the accuracy, particularly for LC chocolate consumption, which may have resulted in a significant reduction in the rate of correct responses. Conversely, the consumption of HC chocolate may have resulted in maintaining accuracy while maintaining reaction time in situations where cognitive exertion was being addressed at full effort.

### 4.2 Increased alertness and concentration suggested by subjective feelings and autonomic nerve function

Changes in subjective feelings and autonomic nerve function were further interpreted. It has previously been shown that the consumption of high-cacao polyphenols and dark chocolate may reduce subjective fatigue. Acute intake of high-cacao polyphenol has been demonstrated to improve performance on continuous cognitive tasks and reduce task-related subjective fatigue [19], and more recently, daily dark chocolate consumption has been shown to reduce sub-chronic fatigue states [41]. In contrast, this study found that the increase in total and mental fatigue from round 1 to round 2 was statistically significant for both HC and LC chocolate consumption. However, increases in physical fatigue and boredom were significant only with LC chocolate consumption and not with HC chocolate consumption. These results suggest that the consumption of HC chocolate contributes to maintaining a high level of alertness, regardless of the increase in fatigue. This may be supported by the results for the autonomic nerve function, where the ratio of LF to HF power was more dominant from round 1 to round 2 with HC chocolate consumption.

### 4.3 Limitations

This study had some limitations. Chocolate contains various bioactive ingredients in addition to polyphenols, such as theobromine, caffeine, fiber, and aroma components. The concentrations of these ingredients differed between the two test foods used in this study; thus, the contributions of these ingredients are unknown.

Another limitation is the lack of clear differences in the effects of the two types of chocolate on cognitive performance through direct comparison. One possible reason for this is the small amount of polyphenol in the HC chocolate. Some previous studies have set the intake of polyphenols, such as cacao flavanols, at 900 mg [11], while others have set it at 445 mg [10]. In this study, the amount of flavanols was converted to approximately 182 mg (HC chocolate) and 63 mg (LC chocolate), according to the ratio of epicatechin and flavanols in a previous study [19]. The flavanol content in HC chocolate was low compared to that in previous studies. Another reason is the small dose contrast between cacao polyphenols in HC and LC chocolate; the amount of cacao polyphenols in LC chocolate (211.7 mg) was lower than that in HC chocolate (635 mg) but may have had a small effect on maintaining cognitive performance. Additionally, a recent systematic review [42] summarized studies on the effects of a single serving of cocoa polyphenol-fortified chocolate and reported improved cognitive function in a study of approximately 30 participants. Therefore, it could be inferred that the small sample size of this study may have limited significant differences for direct comparison. Future studies with larger sample sizes of 30 or more may provide more reliable results. The possible effect of chocolate consumption habits on the maintenance of cognitive performance during long cognitive tasks should also be considered. Although it can be inferred that the participants in this study had prior experience with dark chocolate, no information regarding their chocolate consumption habits was obtained. Moreover, to the best of our knowledge, it is unknown whether cacao polyphenol intake habits result in tolerance to cacao polyphenols; therefore, future research should clarify how chocolate consumption habits affect cognitive performance maintenance effects.

## 5 Conclusion

While it has long been known that dark chocolate affects cognitive function, the findings of this behavioral study with 18 middle-aged individuals suggest that a single consumption of chocolate with a high concentration of cacao polyphenol contributes to the maintenance of cognitive performance and concentration during continuous and demanding tasks.

## Conflict of interest

This study was performed jointly by RIKEN and Meiji Co., Ltd. Yasuyoshi Watanabe holds the position of joint research chair, and Akihiro Sasaki is a leading researcher in joint research. Yasuyoshi Watanabe and Akihiro Sasaki received funding from Meiji Co., Ltd for this study. Chisato Oba, Kentaro Nakamura, and Midori Natsume are employees of Meiji Co., Ltd., which provided the chocolate used in this study. Kei Mizuno, Yusuke Morito, Kyosuke Watanabe, and Emi Yamano declare no competing interests.

## Funding information

This study was funded by Meiji Co, Ltd.

## Data availability statement

Data will be made available on request.

## Ethics statement

This study was reviewed and approved by the Ethics Committees of RIKEN Center for Biosystems Dynamics Research (RIKEN BDR) (approval No. RIKEN-K2-2018-09) and Meiji Co. Ltd. (approval No. 165). All participants provided informed consent to participate in the study.

## CRediT authorship contribution statement

**Akihiro Sasaki:** Writing – review & editing, Writing – original draft, Visualization, Project administration, Methodology, Investigation, Funding acquisition, Formal analysis, Data curation. **Kei Mizuno:** Writing – review & editing, Conceptualization. **Yusuke Morito:** Writing – review & editing, Formal analysis. **Chisato Oba:** Writing – review & editing, Data curation, Conceptualization. **Kentaro Nakamura:** Writing – review & editing. **Midori Natsume:** Writing – review & editing, Methodology, Conceptualization. **Kyosuke Watanabe:** Writing – review & editing, Investigation. **Emi Yamano:** Writing – review & editing, Investigation. **Yasuyoshi Watanabe:** W.

## Declaration of competing interest

The authors declare the following financial interests/personal relationships which may be considered as potential competing interests:Akihiro Sasaki has patent pending to the Japan Patent Office. Kei Mizuno has patent pending to the Japan Patent Office. Yasuyoshi Watanabe has patent pending to the Japan Patent Office. Chisato Oba has patent pending to the Japan Patent Office. Kentaro Nakamura has patent pending to the Japan Patent Office. Midori Natsume has patent pending to the Japan Patent Office.
