# Supplementary material for: The effects of dark chocolate on cognitive performance during cognitively demanding tasks: A randomized, single-blinded, crossover, dose-comparison study
Source: Heliyon. 2024 Jan 11;10(2):e24430. doi: 10.1016/j.heliyon.2024.e24430 (PMC10803911; doi:10.1016/j.heliyon.2024.e24430)
Supplement: Multimedia component 1 [file mmc1.docx]

Supplementary information 1.

Questionnaire for screening

1. Questions about the intake of pharmaceuticals, quasi-drugs, and dietary supplements
   1. I have consumed pharmaceuticals, quasi-drugs, or dietary supplements on at least 5 days per week during the month prior to the screening test. [Yes / No]
   2. I plan to consume the abovementioned on at least 5 days per week during the study period. [Yes / No]
2. Questions about serious diseases such as cranial nerve disease, liver disease, kidney disease, cardiac disease, circulatory disease, malignant tumor
   1. I currently have one of these diseases. [Yes / No]
   2. I have had one of these diseases or conditions. [Yes / No]
3. Questions about seizures due to neurological diseases such as loss of consciousness, coma, convulsions.
   1. I currently have one of these diseases. [Yes / No]
   2. I have had some of these diseases or conditions. [Yes / No]
4. I have food allergies. [Yes / No]
5. I cannot consume bitter chocolate (dark chocolate). [Yes / No]

Note: Bitter (dark) chocolate is defined as chocolate with 40–60% cocoa liquor without milk (dairy), chocolate with a low sugar and dairy content and high bitterness, or low sugar chocolate with 70–90% cocoa content.

1. I consume excessive alcohol (average of 60 g/day or more). [Yes / No]
2. I have a smoking habit. [Yes / No]
3. I work day/night shifts and plan to work night shifts during the study period.

[Yes / No]

1. Questions about your participation in clinical research outside of this study.
   1. I have participated in another study within 1 month prior to providing consent to participate in this study. [Yes / No]
   2. I plan to participate in another clinical research study, during the same study period, after providing consent to participate in this study and during the study period. [Yes / No]
2. Questions about pregnancy (women only)
   1. I am or possibly am currently pregnant. [Yes / No]
   2. I am currently breastfeeding. [Yes / No]
   3. I am planning or hoping to become pregnant during the study period. [Yes / No]

# Supplementary information 2.

# Questionnaire for subjective feelings

Please describe your current condition with a line that intersects the 100 mm horizontal line segment vertically.

Note: The center of the line does not necessarily indicate a “normal” condition.

■ In (1)－(6), the right side is negative feelings.

（1）Total fatigue

　　　　　　　　　　　　 None at all 　　　　　　　　　　　　　　　　　　　　 　 Largest ever

（2）Mental fatigue

　　　　　　　　　　　　 None at all 　　　　　　　　　　　　　　　　　　　　 　 Largest ever

（3）Physical fatigue

　　　　　　　　　　　　 None at all 　　　　　　　　　　　　　　　　　　　　 　 Largest ever

（4）Stress

　　　　　　　　　　　　 None at all 　　　　　　　　　　　　　　　　　　　　 　 Largest ever

（5）Boredom

　　　　　　　　　　　　 None at all 　　　　　　　　　　　　　　　　　　　　 　 Largest ever

（6）Sleepiness

　　　　　　　　　　　　 None at all 　　　　　　　　　　　　　　　　　　　　 　 Largest ever

■In (7)－(12), the right side is positive feelings.

（7）Motivation

　　　　　　　　　　　　 None at all 　　　　　　　　　　　　　　　　　　　　 　 Largest ever

（8）Healing

　　　　　　　　　　　　 None at all 　　　　　　　　　　　　　　　　　　　　 　 Largest ever

（9）Enjoyment

　　　　　　　　　　　　 None at all 　　　　　　　　　　　　　　　　　　　　 　 Largest ever

（10）Relaxation

　　　　　　　　　　　　 None at all 　　　　　　　　　　　　　　　　　　　　 　 Largest ever

（11）Concentration

　　　　　　　　　　　　 None at all 　　　　　　　　　　　　　　　　　　　　 　 Largest ever

（12）Willingness

　　　　　　　　　　　　 None at all 　　　　　　　　　　　　　　　　　　　　 　 Largest ever
